# Noninvasive Positive Pressure Ventilation in Patients with Acute Respiratory Failure Secondary to Acute Exacerbation of Chronic Obstructive Pulmonary Disease

**DOI:** 10.7759/cureus.5820

**Published:** 2019-10-01

**Authors:** Sheeba F Ansari, Mubeen Memon, Naveed Brohi, Amber Tahir

**Affiliations:** 1 Internal Medicine, Liaquat University of Medical and Health Sciences, Jamshoro, PAK; 2 Pulmonology, Civil Hospital, Jamshoro, PAK; 3 Pulmonology, Jinnah Postgraduate Medical Centre Hospital, Karachi, PAK; 4 Internal Medicine, Dow University of Health Sciences, Karachi, PAK

**Keywords:** acute exacerbation of copd, acute respiratory failure, chronic pulmonary obstructive disease, noninvasive ventilation, intubation, invasive ventilation, mechanical ventilation, predictors of mortality, mortality rate

## Abstract

Introduction

Acute exacerbation of chronic obstructive pulmonary disease (AECOPD) is a leading cause of poor quality of life and mortality in developing countries. Noninvasive positive pressure ventilation (NIPPV) remains the first-line intervention in hospitalized patients with acute respiratory failure (ARF) due to AECOPD. However, NIPPV may fail in some patients. This study was conducted to assess the frequency of NIPPV failure and clinical parameters and outcomes in AECOPD patients with failed NIPPV and their conversion to invasive positive pressure ventilation (IPPV).

Methods

This prospective observational study was conducted in the pulmonology unit of a tertiary care hospital in Pakistan. AECOPD patients with ARF who were candidates of NIPPV were included after securing informed consent. Their demographic characteristics, clinical parameters, and in-hospital outcomes were recorded on a semi-structured proforma. For statistical analysis, SPSS software version 22.0 for Windows (IBM, Armonk, NY) was used.

Results

With 24 hours of NIPPV, 73 (70.2%) patients improved and the remaining 31 (29.8%) were shifted to IPPV. Patients in the IPPV group had higher systolic blood pressure (BP) [133.8 mmHg (±21.2) vs. 121.1 mmHg (±8.3); probability value (p): <0.000] and lower diastolic BP [68.7 mmHg (±13.4) vs. 76.2 mmHg (±10.8); p: 0.003]. Their pH was more acidic [7.20 (±0.13) vs. 7.42 (±0.01); p: <0.000], heart rates were high [131.1 (±10.5) vs. 100.2 (±7.5); p: <0.000], and the percentage of oxygen saturation was low [90.7 (±3.0) vs. 93.4 (±4.5); p: 0.004]. Patients who were managed on NIPPV throughout their hospital stay required respiratory support for fewer days [3.2 (±1.3) vs. 4.1 (±1.8); p: 0.005], and their hospital stay was shorter [3.5 (±1.2) vs. 5.3 (±2.5) days; p: <0.000]. Mortality rate in the NIPPV group was significantly lower (1.4% vs. 12.9%; p: 0.01).

Conclusions

Deranged blood pressure, increased heart rate, acidemia, and a low percentage of oxygen saturation are crucial clinical and biochemical parameters that can predict the success of NIPPV with 24 hours of therapy in patients with AECOPD and secondary ARF. Patients who do not improve with 24 hours of NIPPV therapy usually have poor in-hospital outcomes including mortality.

## Introduction

In cases where the damaged respiratory system fails to exchange enough gases, mechanical ventilation must be provided to the patient. Depending on the clinical and biochemical status of the patient, invasive or noninvasive ventilation may be provided [1]. Noninvasive positive pressure ventilation (NIPPV) is provided through a nasal or a face mask at a controlled rate. Invasive positive pressure ventilation (IPPV) is reserved for critical patients and may require endotracheal intubation. The major advantage of NIPPV is that it allows for ventilation support without interfering with the anatomy of the upper airway; however, the use of intubation becomes mandatory in critically ill patients [2].

Chronic obstructive pulmonary disease (COPD) is a major respiratory illness that has a profound impact on public health. World Health Organization (WHO) projects COPD to pose the fifth-highest global disease burden by 2020 [3]. COPD has a 6.2% prevalence in the Asia-Pacific region; of these, 46% has at least one incidence of acute exacerbation of CODP (AECOPD), and 19% of AECOPD cases require hospital admission [4]. AECOPD may be triggered by an infectious etiology or discontinuation of COPD medications or precipitated by congestive heart failure or pulmonary embolism. The triggers may go unidentified in some cases [5].

Over the past few years, various instances have been documented in the literature showing favorable outcomes of utilizing NIPPV therapy in acute respiratory failure (ARF) secondary to AECOPD. These outcomes have been measured in terms of reduced mortality rate, need for emergency endotracheal intubation, duration of hospital stay, and rate of complications [2,6-8]. The efficiency of NIPPV therapy in ARF patients secondary to AECOPD has been documented from Pakistan as well [9]. In comparison to IPPV, NIPPV patients had a significantly shorter duration of ventilation, intensive care unit (ICU) stay, and hospital stay. The failure rate of NIPPV was 20% [9]. In-ICU mortality was significantly lower in the NIPPV group; however, post-ICU in-hospital mortality was comparable between the two groups.

Despite promising outcomes of NIPPV in ARF patients, its utilization is limited to only those patients who are alert, conscious, and cooperative to therapy. AECOPD patients with severe respiratory distress may be delirious or irritated and may have a Glasgow Coma Scale (GCS) score of less than 10. They may be hemodynamically unstable or may have concomitant cardiac arrest necessitating emergency intubation and ventilation [10]. NIPPV cannot be administered as first-line therapy in these patients. Furthermore, patients initially managed on NIPPV may deteriorate and require invasive ventilation during the course of their hospital stay. The rate of NIPPV failure has been reported to be 13-53% in the literature [9,11-13].

Some studies have compared the clinical and biochemical parameters and outcomes in patients with ARF secondary to AECOPD managed via invasive and noninvasive ventilation [2,7,9,10]. However, few studies have evaluated the patient characteristics and factors leading to the failure of NIPPV in these patients [12]. Hence, this study was conducted to evaluate the frequency of NIPPV failure and assess the clinical and biochemical parameters and outcomes in AECOPD patients with failed NIPPV and subsequent initiation of IPPV.

## Materials and methods

This prospective observational study was conducted in the pulmonology unit of a tertiary care hospital in Pakistan. The study was performed from January 1, 2019 to June 30, 2019. We conducted the study after gaining approval from the institutional review board.

Patients included in the study were known cases of COPD and were admitted with acute exacerbation and acute respiratory failure. AECOPD was defined as sudden worsening breathlessness, cough, increased sputum production, and change in sputum color [14]. ARF was categorized as hypercapnia (PaCO_2_: >50 mmHg; pH: <7.30) and hypoxemia (PaO_2_: <60 mmHg) [15]. Only those patients who were eligible for noninvasive positive pressure ventilation (NIPPV) were included. The patients with any two of the following symptoms were deemed eligible for NIPPV: (i) moderate-to-severe respiratory distress with breathlessness and utilization of accessory muscles along with paradoxical abdominal movement (ii) respiratory rate (RR) of >25 breaths/minute (iii) moderate-to-severe acidosis (pH: 7.30-7.35) and hypercapnia (PaCO_2_: 45-60 mmHg) (iv) moderate-to-severe hypoxemia (PaO_2_: <60 and PaCO_2_: <45 mmHg). Patients who needed intubation and invasive ventilation immediately, such as those with severe respiratory distress, severe acidemia, respiratory arrest, altered mental status, and those who were hemodynamically or metabolically unstable, were excluded [3]. The clinical and biochemical parameters of all patients were assessed at two hours and 24 hours from the initiation of NIPPV. Those who did not improve and/or deteriorated within/at 24 hours of NIPPV and required immediate invasive ventilation via endotracheal tube were classified into “invasive ventilation group”, and those who improved on NIPPV were classified into “noninvasive ventilation group”. Patients who deteriorated and required invasive ventilation much after 24 hours of initiation were excluded to reduce bias.

A semi-structured proforma was created to record patient demographic and clinical parameters. Patient age, gender, comorbidity status, duration of COPD, and previous COPD-related hospitalizations were recorded. The clinical parameters included in this study were RR, heart rate (HR), blood pressure (BP), oxygen saturation (SpO_2_), arterial blood pH, and GCS score. Outcomes were assessed in terms of duration of respiratory support, duration of hospital stay, and in-hospital mortality.

For statistical analysis, SPSS software version 22.0 for Windows (IBM, Armonk, NY) was used. Categorical variables were presented as frequencies and percentages. Continuous variables were presented as mean and standard deviation (SD). A chi-squared test was used for categorical variables, and an independent student t-test was used for quantitative variables. A p-value of ≤0.05 was considered significant.

## Results

The study included 104 patients who fulfilled the inclusion criteria. There were more males than females (80% vs. 20%). The mean age of the study sample was 39.3 (±18.4) years. Most of the patients (52%) had been suffering from COPD for more than five years. The mean duration of COPD was 4.4 (±1.8) years. There were some patients with respiratory comorbidities such as tuberculosis (18%) and asthma (10%). Cardiometabolic comorbidities were more common. All baseline characteristics are summarized below (Table 1).

**Table 1:** Baseline characteristics of the patients at the time of admission (n = 104) *Mean (±standard deviation): 39.3 years (±18.4) COPD: chronic obstructive pulmonary disease; AECOPD; acute exacerbation of chronic obstructive pulmonary disease

Baseline characteristics	Number of patients (%)
Gender	
Male	83 (79.8%)
Female	21 (20.2%)
Age*	
Less than 40 years	23 (22.1%)
40-60 years	42 (40.4%)
Above 60 years	39 (37.5%)
Duration of COPD	
Less than 2 years	11 (10.5%)
2-5 years	39 (37.5%)
More than 5 years	54 (51.9%)
Previous hospitalization for AECOPD	
Never	29 (27.8%)
Once	62 (59.6%)
More than once	13 (12.5%)
Co morbidity status	
Tuberculosis	19 (18.3%)
Asthma	11 (10.5%)
Other chronic respiratory diseases	08 (7.6%)
Cardiovascular disease	28 (26.9%)
Metabolic disease	38 (36.5%)

The clinical characteristics of the patients at two hours of NIPPV are summarized below (Table 2).

**Table 2:** Clinical parameters when assessed after two hours of initiation of treatment (n = 104) SpO_2_: peripheral capillary oxygen saturation

Clinical Parameters	Mean (± Standard Deviation)
Systolic blood pressure, mmHg	134.5 (±19.7)
Diastolic blood pressure, mmHg	70.3 (±20.5)
Respiratory rate per minute	25.5 (±8.5)
Glasgow Coma Scale score	12 (±3)
Arterial pH	7.21 (±0.11)
SpO2_,_ %	84.6 (±8.2)
Heart rate per minute	123.2 (±13.6)

The patients were again assessed at 24 hours of NIPPV therapy. At this stage, patients who had improved clinically as well as biochemically were separated from those who either had not improved or deteriorated. Seventy three (70.2%) patients improved and the remaining 31 (29.8%) were shifted to invasive ventilation. Patients in the invasive ventilation group had higher systolic BP [33.8 (±21.2) vs. 121.1 (±8.3) mmHg; p: <0.000] and lower diastolic BP [68.7 (±13.4) vs. 76.2 (±10.8) mmHg; p: 0.003); their pH was more acidic [7.20 (±0.13) vs. 7.42 (±0.01); p:<0.000]; heart rates were higher [131.1 (±10.5) vs. 100.2 (±7.5); p: <0.000]; and oxygen saturation percentage was lower [90.7 (±3.0) vs. 93.4 (±4.5); p: 0.004). GCS score was not considered a valid parameter for intubated and ventilated patients as they were sedated. RR was not deemed a valid parameter for intubated and ventilated patients as it was determined by the ventilator parameters and not by any effort of the patients. All other parameters are compared below (Table 3).

**Table 3:** Clinical parameters at 24 hours in patients improved with noninvasive positive pressure ventilation and patients shifted to invasive ventilation (n = 104) *standard deviation SpO_2_: peripheral capillary oxygen saturation

Clinical parameters	Noninvasive ventilation group (n = 73)	Invasive ventilation group (n = 31)	Probability value
Systolic blood pressure, mmHg	121.1 (±8.3)*	133.8 (±21.2)*	<0.000
Diastolic blood pressure, mmHg	76.2 (±10.8)*	68.7 (±13.4)*	0.003
Arterial pH	7.42 (±0.01)*	7.20 (±0.13)*	<0.000
Heart rate per minute	100.2 (±7.5)*	131.1 (±10.5)*	<0.000
SpO_2,_ %	93.4 (±4.5)*	90.7 (±3.0)*	0.004

Outcomes in patients who recovered with NIPPV only were compared with those who required intubation and ventilation on three parameters. Patients who improved on NIPPV required respiratory support for a mean duration of 3.2 (±1.3) days as compared to 4.1 (±1.8) days in patients who were eventually intubated (p: 0.005). Similarly, the duration of hospital stay was longer for intubated and ventilated patients as compared to those who improved on NIPPV only [5.3 (±2.5) vs. 3.5 (±1.2) days; p: <0.000]. There was only one (1.4%) death reported in the NIPPV group as compared to 4 (12.9%) in invasive ventilation group (p: 0.01). All outcomes are summarized below (Table 4).

**Table 4:** In-hospital outcomes in patients improved with noninvasive positive pressure ventilation and patients shifted to invasive ventilation (n = 104) *standard deviation

Patient outcome	Noninvasive ventilation group (n = 73)	Invasive ventilation group (n = 31)	Probability value
Duration of respiratory support, days	3.2 (±1.3)*	4.1 (±1.8)*	0.005
Duration of hospital stay, days	3.5 (±1.2)*	5.3 (±2.5)*	<0.000
In-hospital mortality	1 (1.4%)	4 (12.9%)	0.01

## Discussion

The rate of NIPPV failure and subsequent immediate endotracheal intubation was high in this study. Failure and conversion to IPPV were predicted by unimproved BP, HR, oxygen saturation, and arterial pH at 24 hours of NIPPV therapy. Patients who were managed on NIPPV throughout the hospital stay required respiratory support for significantly fewer days, and their mean hospital stay was significantly shorter than patients who were converted to IPPV. The mortality rate was significantly higher in patients with failed NIPPV and immediate intubation.

Meeder et al. have reported a 30% NIPPV failure in ARF patients. They reported longer ICU stay and lower survival rates in these patients as compared to those with successful NPPV. They concluded that the hospital outcomes of NIPPV-failed and primarily intubated patients were similar [7]. In a multicenter database study conducted over 15 years (1997-2011), Schnell et al. reported that the utilization of NIPPV for patients with ARF needing ventilator support has increased from 29% to 42%; failure of NIPPV remains an independent risk factor for mortality [16]. In acute respiratory failure due to AECOPD or any other etiology, NIPPV has failed as the first-line intervention in as many as 13-53% patients [9,11-13]. And the mortality rate has been higher in patients immediately intubated after NIPPV failure [13,17]. In a comparative study from India, Bhattacharyya reported a 10% (n = 4/39) failure of NIPPV in COPD and subsequent intubation. Two of these patients died (n = 2/4; 50%) as compared to 36% (n = 4/11) mortality in NIPPV failure and intubation in non-COPD patients [8].

In an observational study from Egypt, 94% of cases of ARF due to AECOPD were successfully managed with NIPPV. Failure was associated with lower GCS score, increased HR, and reduced systolic as well as diastolic BP. Independent predictors of NIPPV failure included advanced age (65+ years), RR rate of ≥35 per minute, pH of <7.26, and white blood cell count of either ≥20,000 or <4,000 [18]. The failure rate was very high and increased HR and RR and acidemia were also associated with NIPPV failure in our study. In another retrospective cohort, male gender and deranged pH even after 72 hours of NIPPV therapy in patients admitted for the first time with ARF secondary to AECOPD were independent prognostic factors for mortality at one year [19]. The work of Fan and colleagues deduced weak cough, high disease severity, and malnutrition as independent risk factors for failure of NIPPV in AECOPD patients. The failure rate reported in their work is 21%. NIPPV-failed patients stayed longer in ICU, had higher ICU and hospital costs, and higher in-hospital mortality [20]. Soliman and colleagues reported a 28.5% failure rate of NIPPV and immediate intubation in AECOPD patients at 24 hours from the initiation of therapy [21]. This is comparable to our results (30%) at 24 hours. They predicted NIPPV failure in patients with high BMI, lower initial pH, increased RR, higher PaCO2, and insignificant response to NIPPV after one hour [21].

In most of the instances where NIPPV is compared with IPPV in terms of outcomes, the patients had indications for either modality [2,9,10]. Patients managed via IPPV were serious enough to not be left on NIPPV alone. However, in this study, all patients were moderate-to-severely ill and were candidates of NIPPV at the start of the study. We believe it to be a major strength of this study. We assessed invasive ventilation only in patients where NIPPV failed and immediate intubation was indicated; hence, the comparison is more precise. However, this study might not have included all predictors of outcome, such as the physician’s decision about intubation. Due to resource constraints, some biochemical predictors may have been missed out. NIPPV failure was considered at 24 hours from the initiation of therapy only, not before or after that. This may have produced more precise data but also resulted in the exclusion of potential cases. Where this data indicates an association, the results cannot be generalized and must only be considered as a hypothesis indicating the need to conduct randomized controlled trials with a more robust methodology to establish the authenticity of clinical and biochemical parameters at 24 hours of NIPPV in predicting its failure and subsequent immediate intubation.

## Conclusions

Noninvasive ventilation in acute respiratory failure due to AECOPD has shown great success rate. However, in some patients, its failure is inevitable. Failure of NIPPV and subsequent immediate intubation could be predicted by unimproved BP, HR, oxygen saturation, and arterial pH at 24 hours from the initiation of NIPPV therapy. Patients who did not improve with 24 hours of NIPPV therapy had poor in-hospital outcomes, including death. Invasive ventilation should be considered in patients who do not respond to noninvasive ventilation even after 24 hours. There is a pressing need to conduct randomized controlled trials with a more robust methodology to establish the authenticity of clinical and biochemical parameters at 24 hours of NIPPV in predicting its failure and subsequent immediate intubation.

## References

[1] Kasper D, Fauci A, Hauser S, Longo D, Jameson J, Loscalzo J (2015). Harrison's principles of internal medicine, 19th ed. Harrison's principles of internal medicine. 19th ed. Philadelphia: McGraw-Hill Education.

[2] Maleh VA, Monadi M, Heidari B, Maleh PA, Bijani A (2016). Efficiency and outcome of non-invasive versus invasive positive pressure ventilation therapy in respiratory failure due to chronic obstructive pulmonary disease. Caspian J Intern Med.

[3] Pauwels RA, Buist AS, Calverley PM, Jenkins CR, Hurd SS, GOLD Scientific Committee (2001). Global strategy for the diagnosis, management, and prevention of chronic obstructive pulmonary disease: NHLBI/WHO Global Initiative for Chronic Obstructive Lung Disease (GOLD) workshop summary. Am J Respir Crit Care Med.

[4] Lim S, Lam DC, Muttalif AR (2015). Impact of chronic obstructive pulmonary disease (COPD) in the Asia-Pacific region: the EPIC Asia population-based survey. Asia Pac Fam Med.

[5] Ko FW, Chan KP, Hui DS, Goddard JR, Shaw JG, Reid DW, Yang IA (2016). Acute exacerbation of COPD. Respirology.

[6] Osadnik CR, Tee VS, Carson‐Chahhoud KV, Picot J, Wedzicha JA, Smith BJ (2019). Non‐invasive ventilation for the management of acute hypercapnic respiratory failure due to exacerbation of chronic obstructive pulmonary disease. Cochrane Database Syst Rev.

[7] Meeder AM, Tjan DH, van Zanten AR (2016). Noninvasive and invasive positive pressure ventilation for acute respiratory failure in critically ill patients: a comparative cohort study. J Thorac Dis.

[8] Bhattacharyya D (2017). Noninvasive ventilation in acute respiratory failure: experience from a tertiary care hospital in India. Am J Respir Crit Care Med.

[9] Devi P, Raja R, Kumar R, Shah A, Ansari SI, Kumar B (2019). Invasive versus non-invasive positive pressure ventilation in chronic obstructive pulmonary disease complicated by acute respiratory failure. Cureus.

[10] Lindenauer PK, Stefan MS, Shieh MS, Pekow PS, Rothberg MB, Hill NS (2014). Outcomes associated with invasive and noninvasive ventilation among patients hospitalized with exacerbations of chronic obstructive pulmonary disease. JAMA Intern Med.

[11] Raju BM, Jotkar S, Prathyusha M, Goswami S, Dube M, Singh A (2018). Effectiveness of non-invasive positive pressure ventilation for acute exacerbation of chronic obstructive pulmonary disease. Int J Clin Trials.

[12] Kumar A, Kumar A, Rai K, Ghazal S, Rizvi N, Kumar S, Notani S (2015). Factors leading to poor outcome of noninvasive positive pressure ventilation in acute exacerbation of chronic obstructive pulmonary disease. J Acute Dis.

[13] Mosier JM, Sakles JC, Whitmore SP (2015). Failed noninvasive positive-pressure ventilation is associated with an increased risk of intubation-related complications. Ann Intensive Care.

[14] Crisafulli E, Barbeta E, Ielpo A, Torres A (2018). Management of severe acute exacerbations of COPD: an updated narrative review. Multidiscip Respir Med.

[15] Shebl E, Burns B (2019). Respiratory Failure. https://www.ncbi.nlm.nih.gov/books/NBK526127/.

[16] Schnell D, Timsit JF, Darmon M (2014). Noninvasive mechanical ventilation in acute respiratory failure: trends in use and outcomes. Intensive Care Med.

[17] Carrillo A, Gonzalez-Diaz G, Ferrer M (2012). Non-invasive ventilation in community-acquired pneumonia and severe acute respiratory failure. Intensive Care Med.

[18] Aziz AO, El Bary IM, Fattah MT, Magdy MA, Osman AM (2017). Effectiveness and safety of noninvasive positive-pressure ventilation in hypercapnia respiratory failure secondary to acute exacerbation of chronic obstructive pulmonary disease. Egypt J Bronchol.

[19] Sprooten RT, Janssen MT, Braeken DC, Rohde GG (2017). Response to non-invasive ventilation as a predictor for mortality in patients with acute respiratory failure due to acute exacerbation of COPD. Eur Respir J.

[20] Fan L, Zhao Q, Liu Y, Zhou L, Duan J (2014). Semiquantitative cough strength score and associated outcomes in noninvasive positive pressure ventilation patients with acute exacerbation of chronic obstructive pulmonary disease. Respir Med.

[21] Soliman MA, El-Shazly MI, Soliman YM, Mostafa AI (2014). Effectiveness of non invasive positive pressure ventilation in chronic obstructive pulmonary disease patients. Egypt J Chest Dis Tuberc.

